# Management of Peritonsillar Abscess Within a Local Emergency Department: A Quality Analysis Study

**DOI:** 10.7759/cureus.17545

**Published:** 2021-08-29

**Authors:** Briana K Ortega, Spencer Short, Bryan G Kane, Robert Dedio

**Affiliations:** 1 Emergency Medicine, Lehigh Valley Health Network, Allentown, USA; 2 Otolaryngology - Head and Neck Surgery, Lehigh Valley Health Network, Allentown, USA

**Keywords:** peritonsillar abscess, pta, incision and drainage, emergency department, ct scan

## Abstract

Objective: Peritonsillar abscess (PTA) is the most common deep space infection of the head and neck, affecting thousands of people annually with high treatment costs. The purpose of this project was to determine how in-network emergency departments (EDs) adhere to generally accepted guidelines regarding diagnosis and management of potential PTAs.

Methods: The authors performed a retrospective chart review to identify patients with PTA in five EDs in one year. Information pertaining to diagnostic tests, treatment, and airway status was also collected. Descriptive analysis was used to assess if EDs were consistent with generally accepted guidelines.

Results: Six hundred twenty-one patient records were identified and 140 were included in final analysis. Out of 140 patients, 71 were admitted for inpatient management and 23 were admitted for observation. Of the 46 patients diagnosed and discharged from the ED, 61% received a computerized tomography (CT) scan and only 39% had PTA drainage performed. Four (3%) patients received a point of care ultrasound and a CT scan and no patient received only an ultrasound. Out of all patients, 116/140 received a CT scan and 22 received drainage in the ED. The remainder of these patients either had drainage performed by an otolaryngologist or had no drainage performed. Of the 94 patients admitted for inpatient or observation, 84 received a CT scan and six received drainage by an ED physician. Only 62% of patients were given a penicillin derivative and 29% were given clindamycin, which has no Gram-negative coverage.

Conclusion: One-third of PTA patients were managed within the ED, far less than similar studies. Of these, over 50% received a CT scan and less than 50% had PTA drainage. PTA drainage can improve patients’ symptoms and antibiotic effectiveness. The majority of patients were prescribed a penicillin derivative with or without another antibiotic.

## Introduction

Peritonsillar abscess (PTA) is the most common deep space infection of the head and neck, affecting 45,000 people per year with annual treatment costs of over $150 million [1]. Patients typically present with unilateral pharyngeal discomfort, drooling, and fever [2]. PTAs present similarly to peritonsillar cellulitis; however, management is dependent on differentiation. Differentiation can be done by identification of pus on needle aspiration, which is a rapid and inexpensive method, and concurrently treats a PTA [2,3]. Despite this identification method, computerized tomography (CT) scans are utilized in approximately 5-20% of cases possibly because of the high sensitivity and specificity [4]. However, CT scans result in radiation doses that may increase the rate of cancer to 390 per million persons [5]. Some providers prefer to utilize imaging prior to PTA drainage for fear of carotid artery injury, although this has not been reported in the literature [5].

Patients can be safely monitored within the emergency department (ED) for a few hours after needle aspiration to assess airway function and discharged with a follow-up appointment with their primary care provider [2]. Previous research found that 80% of PTA patients in the United States are treated with antibiotics without PTA drainage, in the ED, and discharged home with low readmission rates [4]. One study comparing incision and drainage (I&D) with local lidocaine versus needle aspiration under general anesthesia found shorter hospital stays and a lower risk of repeated procedures if the patient was treated with an initial I&D [6]. A clear protocol with well-defined admission criteria can aid physicians in assessing and treating PTA [7].

According to the American Family Physicians guidelines and the literature, PTAs should be treated in an outpatient setting with oral antibiotics, drainage, and steroids [2]. Adequate antibiotic coverage should include Gram-negative, Gram-positive, and anaerobic coverage due to PTAs polymicrobial nature, followed by culture results and tailored treatment [1,2]. Antibiotics such as amoxicillin-clavulanate and ceftriaxone are commonly used and can be complemented by vancomycin or linezolid for methicillin-resistant *Staphylococcus aureus* (MRSA) coverage [2,8]. Despite clear guidelines, empiric antibiotic treatment is not consistently followed [9]. Steroids are often prescribed alongside antibiotics to decrease dysphagia, trismus, and time spent in the hospital [10]. Medical therapy has been shown to be equally effective to drainage and resulted in fewer sick days in some patients, lower opioid usage, and decreased soreness [1]. However, I&D exhibits a success rate of 93-95%, and is a relatively inexpensive and simple procedure, making it the most common treatment approach [11,12]. Therefore, the primary objective of this study was to evaluate how a local hospital network’s EDs compared to generally accepted guidelines regarding the diagnosis and management of potential PTAs with respect to attempted drainage, appropriate antimicrobial therapy, and outpatient management. Secondary objectives focused on quality of patient care regarding imaging studies performed, medications prescribed, and appropriate level of care.

This article was previously presented at the American College of Emergency Physicians Conference 2020.

## Materials and methods

Data collection

After Institutional Review Board approval, a retrospective chart review was done utilizing the International Classification of Diseases (ICD)-10 codes. The following ICD-10 codes were used: J36 (peritonsillar abscess), K12.2 (cellulitis and abscess of mouth), J39.1 (other abscess of pharynx), J39.0 (retropharyngeal and pharyngeal abscess), J39.2 (other disease of the pharynx), J02 (acute pharyngitis), and R07.0 (pain in throat). Multiple ICD-10 codes were used to ensure inclusion of all eligible patients. All patients seen within one calendar year from March 2019 to March 2020 were included in the study. Inclusion criteria were a diagnosis of a PTA or peritonsillar cellulitis and presentation at an in-network ED. Exclusion criteria were no PTA or peritonsillar cellulitis diagnosis or presentation at any location other than an in-network ED. After the initial search was completed, all charts were reviewed by an individual chart reviewer to determine eligibility based on the eligibility criteria. Patients with peritonsillar cellulitis were included in an effort to include all cases of PTAs. Imaging studies related to these patients were reviewed to effectively rule in or out PTA. Portions of the electronic medical record reviewed included history, physical examination, diagnostic studies, hospital course, medications given, and readmission within 10 days. Patients were compared to the aforementioned guidelines for PTA treatment. Descriptive analysis was performed using Jeffrey's Amazing Statistics Program (JASP) version 0.9.0.1 (The Netherlands: University of Amsterdam).

## Results

**Figure 1:**
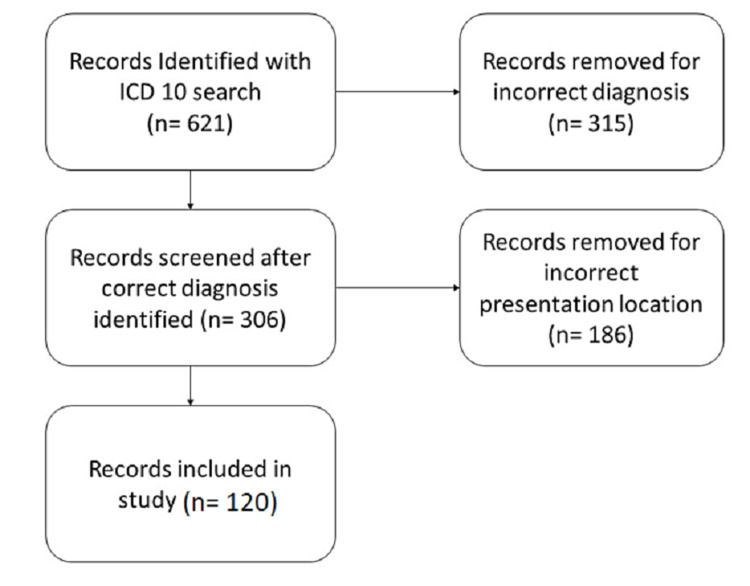
Patient records selected for analysis.

Six hundred twenty-one patients were identified utilizing ICD-10 codes. Of these, 295 patients did not have a PTA or peritonsillar cellulitis diagnosis and 186 patients did not present to an in-network ED and were excluded from the study, leaving 140 patients in the study (Figure 1). Sixty-seven (56%) patients were male and 53 (44%) were female. The average age was 31.3 years (15.74 SD) ranging from two years of age to 90 years of age. The average length of stay was 1.4 days (1.56 SD) ranging from zero days to 11 days (Table 1).

**Table 1:** Patient and location presentation characteristics of patients with a peritonsillar abscess.

Patient and Location Presentation Characteristics		
Age (Mean) - year (SD)	31.7 (16.21)	
Gender, N (%)		
Male		67 (56%)
Female		53 (44%)
Presenting Hospital, N (%)		
Main Hospital		85 (71%)
Secondary Hospital		21 (18%)
Smaller Hospital		14 (11%)
Admission Location, N (%)		
Emergency Department		35 (29%)
Observation Unit		21 (18%)
Inpatient		64 (53%)
Length of Stay (Mean) - D (SD)		1.5 (1.61)

One hundred (71%) patients presented at our primary hospital (a level one trauma center with a separate children’s ED), 61 (61%) were admitted to the inpatient service, and 19 (19%) were held for observation. There were 24 (17%) patients who presented at the level two trauma center hospital, five (21%) were admitted to the inpatient service, and two (8%) were held for observation. There were 16 (12%) patients seen at smaller hospitals within the hospital network five (31%) were admitted to the inpatient service, and two (13%) were held for observation. Out of all patients, 71 (51%) were admitted to the hospital, 23 (16%) were held for observation, and 46 (33%) were discharged from the ED. Patients’ presenting symptoms were documented. The majority, 121 (86%), had either dysphagia, odynophagia, or hoarseness documented in their initial evaluation. Of the patients without recorded dysphagia, odynophagia, or hoarseness, 7/19 (37%) were placed on a nothing by mouth (NPO) diet.

After reviewing the patients’ initial presentation, pertinent portions of their hospital stay were recorded including imaging, consults, PTA drainage, and medications given. All patients’, with a PTA ICD code, imaging results were reviewed, and those that did not have a visible PTA on imaging, 20/140 (14%), were considered to have peritonsillar cellulitis and were not included in analysis. Most patients received some form of imaging, with 94 (78%) patients receiving a CT scan, four (3%) received a point of care ultrasound and CT scan, two (2%) received an x-radiograph, and only 20 (17%) received no imaging. Patients who received a CT scan were more likely to be admitted, with a chi-square p-value of 0.003. Of the same patients, only 58 (52%) had an identified abscess on imaging, 40 (34%) had a potential abscess on imaging, and 18 (16%) had no abscess on imaging (Table 2). In the case that the radiologist could not concretely rule in or out a PTA on imaging, “a potential abscess” was listed within the report.

**Table 2:** Imaging and procedures performed during hospital stay. CT- computerized tomography; PoCUS- point of care ultrasound; ED- emergency department; ENT- ears, nose, and throat; OR- operation room

Imaging Performed, N (%)	
CT Scan	94 (78%)
PoCUS and CT Scan	4 (3%)
X-Ray	2 (2%)
No Imaging	20 (17%)
PTA Drainage Performed, N (%)	
ED Provider	19 (16%)
ENT Provider	25 (21%)
OR Drainage	12 (10%)
ED and ENT Provider	1 (1%)
ED Provider and OR	1 (1%)
No Procedure	62 (51%)

Transferred patients

Nearly a quarter of patients, 22/120 (18%), were transferred from smaller in-network hospitals to the central hospital for PTA management, despite resources available for aspiration, imaging, and overnight observation at the presenting hospital. Of these patients, 10 (45%) were admitted to the intensive care unit (ICU) and 11 (50%) were placed on airway watch. Only one (4%) transferred patient had documented oxygen desaturation and none required nasal or oral airway support. Only 10 (45%) patients received PTA drainage and 12 (55%) of transferred patients received no PTA drainage.

Consults

The majority of patients received one or more consult, 74/120 (62%) PTA patients received only an otolaryngology consult; eight (7%) received otolaryngology and medicine consult for ICU admission; 15 (12%) received otolaryngology and other specialty consult such as anesthesia, infectious disease, or general surgery; and 23 (19%) received no consults.

Procedures done

Only 19 (16%) patients had PTA drainage performed by an ED provider, 25 (21%) had PTA drainage performed by an otolaryngology provider at bedside, one (1%) had attempted PTA drainage performed by an ED and otolaryngology provider, 12 (10%) had PTA drainage by an otolaryngologist in the operating suite, and one (1%) patient had attempted PTA drainage performed by an ED provider and in the operating suite by an otolaryngologist. Most patients, 62 (51%), had not PTA drainage (Table 2).

Throughout their stay, only two (2%) patients experienced an oxygenation desaturation (defined as less than 90% on pulse oximeter). The remaining 118 (98%) patients had no recorded desaturations. Only one (1%) patient received a nasal or oral airway and two (2%) received supplemental oxygen. Additionally, 21 (17%) patients were held in an observation unit.

Medications prescribed

Initial medications and medications prescribed with discharge were extracted from the patients’ charts. The majority of patients, 78/120 (65%), were given a penicillin derivative, seven (6%) were given penicillin and second class of antibiotic, 33 (28%) were given clindamycin, and two (1%) were given no antibiotics throughout their hospital stay. Along with antibiotics, 95 (79%) patients received steroids throughout their stay. Upon discharge, 95 (79%) patients were prescribed a penicillin derivative antibiotic, 22 (18%) were prescribed clindamycin, and three (3%) were not prescribed antibiotics. Only two (29%) patients had received PTA drainage in their original stay and seven (6%) were seen at the hospital within 10 days of discharge.

## Discussion

Over half (53%) of patients presenting for a PTA were admitted to a hospital and 83% of patients received imaging. This management is contrary to current guidelines and recommendations [2]. Current literature reports that 80% of patients with a PTA are discharged from the ED, whereas the institution discharged only 47% of patients presenting to the ED with a PTA [4]. Additionally, only two patients had a documented oxygen desaturation throughout the stay and one patient received a nasal or oral airway. However, 18 patients were admitted to the ICU for fear of airway compromise, although none exhibited progression of disease. Our findings suggest that this level of care is not necessary and contributes to higher healthcare costs with no added quality.

Patients transferred from smaller hospitals in the network to the largest hospital did not benefit from the higher level of care. Of 22 patients, 10 (45%) were admitted to the ICU and 11 were placed on “airway watch”, despite an ENT provider being present at each hospital. Even with the higher level of concern for airway compromise, only one of these patients had an oxygen desaturation and none were given nasal or oral airway support.

However, medical management of PTAs in this hospital network was consistent with existing literature. Steroids are often given to decrease pain, dysphagia, and time in the hospital and are commonly administered with antibiotics [13]. Nearly 80% of patients were given a penicillin derivative; 79% of patients were given steroids during their stay; and nearly 79% of patients were given a penicillin derivative upon discharge, consistent with treatment guidelines for bacteria that commonly cause a PTA [1]. Unfortunately, the readmission rate was 6%, higher than reported in the literature. One reason for this may be absent or delayed drainage of PTA.

I&D is a common treatment for PTAs and was underutilized within this hospital network. Less than half of the patients (38%) underwent I&D at bedside and of the patients that did, only 46% of them had an I&D performed by an ED provider. Three patients had ultrasound-guided drainage by an ED provider. The remaining 54% had an I&D performed by an ENT provider. The theoretical risk of damaging the carotid artery may act as a deterrent for some providers, but has not been reported in the literature [5]. Additionally, over half of patients who received a CT scan had an identified PTA and would have benefited from PTA drainage in the ED. Only four patients had a point of care ultrasound, and all of these patients also had a CT scan. This highlights the underutilization of ultrasound for diagnosis and treatment. Training utilizing ultrasound for PTA drainage has been found to increase a resident's comfort level with PTA management [11]. The ED at the institution is the primary location for a large Emergency Medicine residency program.

Limitations

The limitations of this study relate to retrospective chart review and based upon provider recall. Documentation may not have included all pertinent symptoms or provider reasoning for management decisions. Additionally, this was done as an internal study, and may not be representative of PTA management nationwide. However, this study does contain a large sample size and gives insight to how smaller hospitals with limited resources may manage PTAs.

## Conclusions

Within this study period, management of PTAs was inconsistent with current guidelines based on the literature from several different disciplines. By decreasing the amount of imaging performed for patients with a PTA, performing an I&D within the ED, decreasing the number of patients transferred from smaller hospitals, and decreasing the number of patients admitted to the ICU, significant cost savings can be achieved, and emergency medicine residents would receive greater insight to PTA diagnosis and management. Antibiotic management was consistent with guidelines. Future quality improvement projects could aid in facilitating these changes.
